# Supermarket and Grocery Store–Based Interventions to Promote Healthful Food Choices and Eating Practices: A Systematic Review

**DOI:** 10.5888/pcd10.120156

**Published:** 2013-04-11

**Authors:** Anne L. Escaron, Amy M. Meinen, Susan A. Nitzke, Ana P. Martinez-Donate

**Affiliations:** Author Affiliations: Amy M. Meinen, Wisconsin Department of Health Services, Madison, Wisconsin; Susan A. Nitzke, Ana P. Martinez-Donate, University of Wisconsin–Madison, Madison, Wisconsin.

## Abstract

**Introduction:**

Increasingly high rates of obesity have heightened interest among researchers and practitioners in identifying evidence-based interventions to increase access to healthful foods and beverages. Because most food purchasing decisions are made in food stores, such settings are optimal for interventions aimed at influencing these decisions. The objective of this review was to synthesize the evidence on supermarket and grocery store interventions to promote healthful food choices.

**Methods:**

We searched PubMed through July 2012 to identify original research articles evaluating supermarket and grocery store interventions that promoted healthful food choices. We categorized each intervention by type of intervention strategy and extracted and summarized data on each intervention. We developed a scoring system for evaluating each intervention and assigned points for study design, effectiveness, reach, and availability of evidence. We averaged points for each intervention category and compared the strength of the evidence for each category.

**Results:**

We identified 58 articles and characterized 33 interventions. We found 7 strategies used alone or in combination. The most frequently used strategy was the combination of point-of-purchase and promotion and advertising (15 interventions); evidence for this category was scored as sufficient. On average, of 3 points possible, the intervention categories scored 2.6 for study design, 1.1 for effectiveness, 0.3 for reach, and 2 for availability of evidence. Three categories showed sufficient evidence; 4 showed insufficient evidence; none showed strong evidence.

**Conclusion:**

More rigorous testing of interventions aimed at improving food and beverage choices in food stores, including their effect on diet and health outcomes, is needed.

## Introduction

Obesity, overweight, and health outcomes associated with poor nutrition (1) represent a significant economic and social burden in the United States. Annual medical costs attributed directly to obesity and overweight were estimated at $147 billion in 2008 (2). Public health researchers and practitioners are working to identify evidence-based interventions to promote more healthful eating practices. The Dietary Guidelines for Americans 2010 recommend stronger environmental strategies for improving the population’s eating practices, including interventions to influence food purchasing behaviors in stores (3).

Supermarkets play an important role in food purchasing (4); consumers averaged 2.2 trips per week to the supermarket in 2010 (5). They also represent an optimal setting for interventions aimed at improving food purchase decisions. Supermarket and grocery store interventions are consistent with a social ecological approach (6,7), and the availability of healthful foods in food stores affects consumers’ ability to make healthful dietary choices (4,6). Low-income populations purchase a high proportion of their food as prepared foods and from small stores, which has implications for intervention development (8).

Several reviews on food store interventions have found strong evidence for feasibility but only modest evidence for effectiveness in changing eating behaviors (4,9–11). A review on interventions in small food stores (12) indicated that 9 of 10 studies observed an increase in the number of purchases of targeted foods. The relationship between large food stores and dietary intake has received attention (13). The objective of this review was to review, summarize, and assess the level of evidence on supermarket and grocery store interventions to promote healthful food choices.

## Methods

### Data sources

We searched all years of PubMed for original research articles and qualitative and quantitative reviews (meta-analyses) describing supermarket and grocery interventions that promoted healthful food choices. We used a combination of keywords (“grocery store,” “grocery stores,” “supermarket,” and “supermarkets”) and 1 MeSH term (“health promotion”). An initial search yielded 140 citations dated from the late 1940s through July 2012.

### Study selection

When we included only English-language articles, 134 remained. The first author (A.L.E.) read each title and abstract; if the article was relevant, she read the full text. She narrowed the search to include only original research articles that described community- (those initiated by public health practitioners) and store-based interventions (in which store involvement was described). She excluded clinical screening interventions and controlled marketing field experiments, articles that did not report the targeted outcomes, and other articles that were not within the scope of the review. She reviewed citations in the selected articles and included those articles if they met criteria. As a result, 58 articles published from 1978 through 2012 were included for further analysis; the 58 articles described 33 interventions.

### Data extraction

The first author (A.L.E.) categorized each article according to the intervention described and the strategy or combination of strategies used in the intervention (Figure). The 4 strategies were point-of-purchase (POP) information, pricing, increased availability of healthful foods, and promotion and advertising. These strategies are consistent with those used previously (4).

**Figure:**
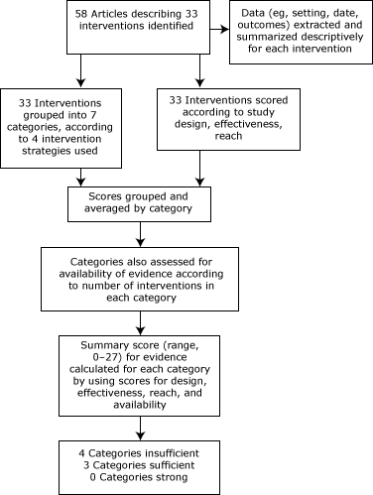
Data extraction and analysis for systematic review on supermarket and grocery store–based interventions to promote healthful food choices and eating practices, United States, 2012.

POP interventions typically entail the use of food demonstrations, taste testing, signs, labels, and other printed materials highlighting healthful food choices or describing recipes with the goal of influencing purchasing decisions toward more healthful options (4). Interventions based on pricing use reduced prices and coupons to promote healthful options (4,10). Interventions based on increased availability work to provide more healthful food choices (4). Promotion and advertising strategies use games, newspaper inserts, multimedia advertising, supermarket tours, and other activities to promote the purchase of more healthful foods (4).

The first author extracted the following data for each intervention: the theory on which the intervention was based (eg, social cognitive), intervention setting, location, year in which an article was published; description of intervention activities and duration; study design (eg, concurrent comparison group, prospective measurement of outcomes); and main outcomes measured. 

The main outcomes were awareness and use, sales data, customers’ knowledge and beliefs (14), preferences, intentions, and process measures (15–17). Awareness and use refers to the percentage of surveyed customers who noticed the intervention materials and believed their purchasing decision was influenced by them. Sales data refers to objective measurements of customers’ purchasing decisions for a category of food or item. Preferences serve as a predictor of target food consumption (18,19). Intentions refer to behavioral intentions to prepare, select, and consume more healthful foods (19). Process measures included reach, dose, and fidelity; reach is the number of target audience members exposed to any component of the intervention (20), dose is the number of times each target audience member was exposed to any intervention component (21), and fidelity is the extent to which an intervention was implemented as planned (15). When interventions reported on fruit and vegetable intake, fat intake, or dietary intake, we also extracted these data. 

We created an assessment schema on the basis of accepted terminology in the *Guide to Community Preventive Services (Community Guide)* (22) and other definitions (20) and categories (15). For each of 3 characteristics — study design, effectiveness, and reach — the first author assigned points to each intervention according to her assessment. Study design was scored as 1, 2, or 3 points, according to the suitability of study design to determine effectiveness (15,22). Greatest suitability (3 points) was defined as a study that had a concurrent comparison group and prospective measurement of outcomes. Moderate suitability (2 points) was defined as retrospective designs or studies that had multiple pre- or postmeasurements but no concurrent comparison group. Least suitability (1 point) was defined as before–after studies that had no comparison group or studies in which outcomes were measured in a single group at the same point in time. We did not assign zero points for study design. 

Effectiveness was scored as 0 to 3 points, according to effectiveness of the intervention’s main outcome measures (eg, awareness and use). Studies reporting a 70% to 100% increase pre- to posttest or between comparison and intervention groups in outcomes (eg, awareness and use) of the intervention were assigned 3 points. Studies reporting a 26% to 69% increase received 2 points. Studies reporting a 1% to 25% increase received 1 point. A score of 0 was assigned if no difference in outcomes was reported between study groups. Most studies reported effectiveness as awareness or use of their intervention (20). If awareness and use were not reported, we assessed the intervention’s main outcome measures (eg, knowledge and beliefs, sales data, preferences, intentions, fruit and vegetable intake, fat intake, dietary intake) and used the same scoring. When an intervention had no effect on awareness or use but had a significant effect on sales data or preferences or intentions, we scored the alternative outcomes. 

Reach was also scored as 0 to 3 points. According to the RE-AIM evaluation framework (20), reach is determined by dividing the number of intervention participants by the number of people in the targeted population. For interventions reaching 70% to 100% of the population, 3 points were assigned; for 26% to 69%, 2 points; for 1% to 25%, 1 point; and for 0%, 0 points.

For each intervention category (eg, POP), we calculated the average number of points for study design, effectiveness, and reach. We used this average as 1 of 2 subscores.

For each intervention category, we assessed the availability of data using the following scoring system. We gave 3 points to categories that included 10 to 30 interventions, 2 points to categories that included 2 to 9 interventions, and 1 point to categories used by only 1 intervention. These points represented the second subscore and functioned as an indicator of the amount of evidence available.

We calculated a summary score (range, 0–27) for each intervention category by multiplying the 2 subscores. We created 3 classes of evidence on the basis of the summary score: insufficient (0–9 points), sufficient (10–18 points), and strong (19–27 points). These classes were based on *Community Guide* designations (22). For all strategies combined, the scores for all 5 categories (ie, availability of evidence, study design suitability, effectiveness, reach, and overall summary) scores were summed and then divided by the number of categories.

## Results

The 33 interventions were implemented in the United States (n = 22), Canada (n = 4) (23–26), and 5 other countries (n = 7) (27–33). In the United States, 4 interventions were in Baltimore, Maryland (34–37); 7 were in the Midwest (38–44). Most interventions (n = 28) were implemented in grocery stores or supermarkets. Only 8 (24,26,28,33,37,39,45,46) targeted racial/ethnic minority populations or populations that had low socioeconomic status. Social cognitive theory (19,47) was the most frequently used theory, undergirding 6 studies; the same research group generated most (28,37,45,46,48) of these. The consumer information processing model was the next most frequently used theory; it was used by 3 studies (34,38,49). Three studies (42,46,50) referenced social marketing theory (51). Nine studies used environmental strategies (23,24,29,34,35,43,52–54) and 19 studies used nutrition education (23,24,29,31–33,35,39–41,43,44,52–58) as both rationale and framework. The most frequently reported outcomes were awareness and use (17 studies) and knowledge and beliefs (18 studies), followed by sales data and process measures.

Interventions were organized into the following categories (Table 1): POP (n = 6); POP and pricing (n = 1); POP and promotion and advertising (n = 15); POP, pricing, and promotion and advertising (n = 4); POP, increased availability of healthful foods, and promotion and advertising (n = 3); POP, pricing, increased availability of healthful foods, and promotion and advertising (n = 2); and pricing and promotion and advertising (n = 2). 

**Table 1:** Studies Published from 1978 Through 2012 on Supermarket and Grocery Store Interventions (N = 33) to Promote Healthful Eating, by Strategy Used^a^

Strategy/Intervention	Summary Data
**Point-of-Purchase**	
**Supermarket information project (** **58** **)**	
Setting, location, and year^b^	2 Intervention supermarkets and 1 control supermarket, Fresno, California, 1978
Activities and duration	Index card brochures distributed in stores but designed for home use; 4 months
Design	Prospective measurement with comparison group
Main outcomes measured	Awareness and use; knowledge and beliefs; sales data
Effectiveness	No changes in sales of targeted items; tripled anticipated response rate for information inquiries; store managers reported customer satisfaction with point-of-purchase
**Supermarket nutrition intervention (** **52** **)**	
Setting, location, and year^b^	3 Supermarkets in suburbs of northern New Jersey, 1990
Activities and duration	Videocassettes, demonstrations, and printed materials^c^; 6 months
Design	Prospective measurement with comparison group
Main outcomes measured	Knowledge and beliefs; sales data
Effectiveness	No effect on food-purchasing behavior
**Nutrition for a Lifetime System (** **59** **–** **61** **)**	
Setting, location, and year^b^	1 Supermarket with control and experimental shoppers, location not available, 1991–2001 (59)
Activities and duration	In-store video programs and printed feedback on intended-purchase function (providing customers with individualized reduced fat and increased complex carbohydrate alternatives); 2 months
Design	Prospective measurement with comparison group
Main outcomes measured	Sales data; intention; preferences; dietary intake; knowledge and beliefs
Effectiveness	25% Greater increase in purchasing of targeted items in intervention compared with control shoppers; interactive nutrition information system used by more than 1,400 (nonpaid) people
**Paint Your Plate (** **23** **)**	
Setting, location, and year^b^	11 Stores had interactive display events; 6 stores had brochures; Greater Sudbury, Canada, 2009
Activities and duration	Interactive display events with public health staff, a display, resources, and food samples vs brochures; 1 month
Design	Prospective measurement with comparison group
Main outcomes measured	Knowledge and beliefs
Effectiveness	Event participants more likely to identify serving size of fruit and vegetables and recommended number of servings of fruits and vegetables
**Educational intervention (** **44** **)**	
Setting, location, and year^b^	4 Experimental and 4 control supermarkets in Twin Cities, Minnesota, 1982
Activities and duration	Printed materials^c^ throughout dairy section; 6 months
Design	Prospective measurement with comparison group
Main outcomes measured	Sales data; knowledge and beliefs
Effectiveness	No clear effect of intervention on nutrition knowledge or sales of targeted products
**Point-of-purchase health information (** **32** **)**	
Setting, location, and year^b^	1 Intervention and 1 control supermarket, Gosen City, Japan, and Tagami Town, Japan, 2011
Activities and duration	Health-related printed materials^c^; 3 months
Design	Prospective measurement with comparison group
Main outcomes measured	Sales data
Effectiveness	18.7 Percentage points greater mean adjusted change in sales of total vegetables in intervention vs control
**Point-of-Purchase and Pricing**	
**Demonstration cancer control project for Iowa farmers (** **38** **)**	
Setting, location, and year^b^	4 Control and 4 intervention supermarkets, small towns in Iowa, 1997
Activities and duration	Printed materials^c^, menus signage; coupons, food demonstrations; 8 months
Design	Prospective measurement with comparison group
Main outcomes measured	Awareness and use; fruit and vegetable intake
Effectiveness	18% to 43% of shoppers were exposed to intervention activities; no evidence of effect on consumption of fruits and vegetables
**Point-of-Purchase and Promotion and Advertising**	
**Special Diet Alert (** **34** **,** **62** **,** **63** **)**	
Setting, location, and year^b^	10 Intervention grocery stores in Washington, DC, and 10 control grocery stores in Baltimore, Maryland, 1992
Activities and duration	Brand-specific shelf markers, take-away information booklets, radio and television spots; 24 months
Design	Prospective measurement with comparison group
Main outcomes measured	Sales data
Effectiveness	Sales of shelf-marketed products increased; media schedule reach was 86% of target population
**M-Fit supermarket shelf-labeling program (** **39** **)**	
Setting, location, and year^b^	18 Supermarkets, Detroit, Michigan, 2000
Activities and duration	Color-coded shelf labels, banners, produce and dairy signs, shopping guide; duration unknown
Design	Single group, same point in time
Main outcomes measured	Awareness and use
Effectiveness	Overall intervention awareness was 28.8%; of those aware of the program, 56% used the program
**Shop for Your Heart (** **24** **,** **64** **–** **66** **)**	
Setting, location, and year^b^	3 Grocery stores and 2 supermarkets, Montreal, Canada, 1995–1998
Activities and duration	Printed materials^c^, guided tours, taste tests, 1-time cholesterol screening event; 4 months
Design	Single group, same point in time
Main outcomes measured	Awareness and use; process measures
Effectiveness	Overall intervention awareness 52%; of those aware of the program, 6% used the program
**Foods for Health (** **35** **,** **67** **)**	
Setting, location, and year^b^	90 Intervention supermarkets, Washington, DC; comparison supermarkets (number not reported), Baltimore, MD, 1983–1986
Activities and duration	Shelf and window signs, banners, printed materials^c^, newspaper advertising, radio spots; 12 months
Design	Prospective measurement with comparison group
Main outcomes measured	Awareness and use; knowledge and beliefs; sales data; process measures
Effectiveness	Improvements in nutrition knowledge in intervention compared with control group
**Food for Health: the Carbohydrate Connection (** **53** **)**	
Setting, location, and year^b^	3 Intervention supermarkets and 2 comparison supermarkets, town in New York State, 1982
Activities and duration	Fact sheet, recipe cards, placard on shelf near target food; newspaper and radio messages; 4 months
Design	Prospective measurement with comparison group
Main outcomes measured	Awareness and use; knowledge and beliefs; dietary intake; sales data
Effectiveness	Improved nutrition knowledge and food use scores among those aware of the campaign; increase in purchase of targeted items
**Eat for Health (** **36** **,** **55** **,** **68** **,** **69** **)**	
Setting, location, and year^b^	>100 Intervention supermarkets, Washington, DC; and 30 control supermarkets, Baltimore, Maryland, 1989–1994
Activities and duration	Shelf labels; information guide; monthly bulletin; signs in produce sections; television, radio, and print advertising; and brief in-store videos; 24 months
Design	Prospective measurement with comparison group
Main outcomes measured	Awareness and use; knowledge and beliefs; fat intake; sales data; process measures
Effectiveness	Modest effect on food purchasing behaviors; improved nutrition knowledge, attitudes, and self-reported food purchasing behaviors; approximately 200,000 people exposed to campaign
**Four Heart Program (** **54** **)**	
Setting, location, and year^b^	3 Supermarkets and 1 grocery store, Pawtucket, Rhode Island, 1990
Activities and duration	Brand-specific shelf labels, signs, printed materials^c^, contests, and blood pressure and cholesterol screening, counseling, and referral events; 48 months
Design	Multiple measurement, no comparison group
Main outcomes measured	Awareness and use
Effectiveness	24% Correctly identified intervention labels; 54% reported being encouraged to purchase identified food. Reach: 1,179 participants and 4,913 population totals; 636 were encouraged to purchase labeled products
**Shop Smart for Your Heart (** **41** **)**	
Setting, location, and year^c^	17 Grocery stores in 3 Minnesota locations: Mankato, Fargo, and Moorhead, 1987
Activities and duration	Shelf labels, taste testing, printed materials^c^, newspaper advertisement, grocery cart inserts; 4 months
Design	Multiple measurement, no comparison group
Main outcomes measured	Awareness and use
Effectiveness	40% Were aware of intervention; 17% to 41% reported that signs influenced their food choices
**Lean Meats Make the Grade (** **40** **)**	
Setting, location, and year^b^	8 Grocery stores in 3 Minnesota locations: Mankato, Fargo, Moorhead, 1988
Activities and duration	Taste testing, printed materials^c^, and package labels; 1 month
Design	Prospective measurement with comparison group
Main outcomes measured	Awareness and use; knowledge and beliefs; sales data
Effectiveness	Greater awareness in intervention (69%) than in comparison (58%) communities; improved nutrition knowledge; increased sales of targeted items
**Low-fat nutrition education and labeling (** **29** **)**	
Setting, location, and year^b^	13 Supermarkets including control and 2 intervention groups, the Netherlands, 2004
Activities and duration	Printed materials^c^, self-help manual, contest, shelf label; 6 months
Design	Prospective measurement with comparison group
Main outcomes measured	Fat intake; knowledge and beliefs; intentions
Effectiveness	No significant effects on total fat intake or psychosocial determinants
**The Good Book of Nutrition (** **56** **)**	
Setting, location, and year^b^	6 Chain supermarkets, Central Florida, 1993
Activities and duration	Taste testing, nutrition information, store advertisements, newspaper advertisements, cookbook, skirting for information booth, printed banners, posters, package stickers, and shelf tags; 1 month
Design	Single group, same point in time
Main outcomes measured	Awareness and use; knowledge and beliefs
Effectiveness	23% Of shoppers were aware of campaign; 3% of shoppers made changes to their diet
**Shop Smart Tour (** **25** **)**	
Setting, location, and year^b^	Supermarket chain in 40 communities, British Columbia, 1993
Activities and duration	Aisle-by-aisle supermarket tour that taught consumers skills in making food choices; follow-up at 3 months
Design	Before and after, no comparison group
Main outcomes measured	Intentions
Effectiveness	23% to 33% of 48 participants intended to alter dietary behavior in targeted direction; posttest purchase of low-fat dairy products, whole grain products, and polyunsaturated spreads was greater than intention to purchase at baseline; posttest purchase of legumes and tofu was less than intention to purchase at baseline
**1% Or Less campaign (** **57** **,** **70** **–** **72** **)**	
Setting, location, and year^b^	8 Intervention stores in Clarksburg, West Virginia, and Bridgeport, West Virginia, and 6 comparison stores in Wheeling, West Virginia, 1998–2005
Activities and duration	Focused message was communicated through paid advertising, public relations activities, and community-based education programs: newspaper, radio, and television advertisements, press conferences, blinded taste tests at supermarkets; signs in dairy case; 2 months
Design	Prospective measurement with comparison group
Main outcomes measured	Preferences; sales data
Effectiveness	Increased sales of targeted items; reach: 231 participants and 257 population totals
**Healthy Kids (** **50** **)**	
Setting, location, and year^b^	1 Intervention store, Roanoke, Virginia, 2012
Activities and duration	Low-to-the-ground kiosk displaying featured food items; 3 months
Design	Multiple measurement, no comparison group
Main outcomes measured	Sales data; awareness and use
Effectiveness	Overall significant increase in proportion of sales of featured items to total store sales
**5 A Day (** **49** **,** **73** **,** **74** **)**	
Setting, location, and year^b^	3 Intervention and 3 comparison stores, supermarket chain in eastern Massachusetts, 1995–2001
Activities and duration	Take-home audiotapes and in-store public service announcements; 1 month
Design	Prospective measurement with comparison group
Main outcomes measured	Knowledge and beliefs; fruit and vegetable intake; intentions; process measures
Effectiveness	Increased knowledge scores in intervention group compared with control group; no effect on fruit and vegetable intake, beliefs, or intentions
**Point-of-purchase, Pricing, and Promotion and Advertising**	
**Tasty and Healthy Campaign (** **30** **)**	
Setting, location, and year^b^	12 Intervention and 6 control butcher shops, Limburg province, the Netherlands, 2006
Activities and duration	Product labels, price reductions, printed materials^c^, mobiles, training employees, television and radio commercials, newspaper advertisements; 3 months
Design	Prospective measurement with comparison group
Main outcomes measured	Dietary intake; awareness and use; intentions
Effectiveness	71% of customers were aware of campaign; no effects on nutrition behavior or intentions
**Kansas LEAN (** **42** **)**	
Setting, location, and year^b^	1 Supermarket, Kansas, 1996
Activities and duration	Prompting by food manufacturer demonstrators consisted of verbal encouragements, taste samples, and coupons; 9.5 hours
Design	Prospective measurement with comparison group
Main outcomes measured	Trained observers counted the number of targeted food items placed by customers into their shopping carts.
Effectiveness	Increased consumer purchases of targeted items
**Lifestyle 2000 Flavour Without Fat (** **27** **)**	
Setting, location, and year^b^	6 Intervention supermarkets, Bunbury, Australia, 1991
Activities and duration	Large mobiles, vinyl striping on produce bins, shelf talkers, cardboard canopies, and barbers’ poles for dairy cabinets included project logo; low-fat dairy products relocated in 1 section of the dairy cabinet; coupons, printed materials^c^, and option to get *Flavour Without Fat* cookbook; low-fat product taste testing; 4 months
Design	Single group, same point in time
Main outcomes measured	Awareness and use; process measures
Effectiveness	52% Were aware of supermarket promotion; of those aware, 22% reported it had influenced their food choices; sales of targeted items increased during demonstration periods
**Guided supermarket tours (** **31** **,** **75** **)**	
Setting, location, and year^b^	9 Supermarkets, The Netherlands, 1996–1998
Activities and duration	Nutrition education tour given by dietitians (and promoted in mass media); printed materials^c^, games played, taste testing; 4 months
Design	Multiple measurement, no comparison group
Main outcomes measured	Knowledge and beliefs; intentions; process measures
Effectiveness	Improved knowledge; 45% of children and 70% of adults intended to buy more low-fat products
**Point-of-Purchase, Increased Availability of Healthful Foods, Promotion and Advertising**	
**Marshall Islands Healthy Stores (** **28** **,** **76** **)**	
Setting, location, and year^b^	12 intervention and 11 control large and small food stores, Majuro Atoll, Republic of the Marshall Islands; 2006–2007
Activities and duration	Newspaper articles, video, and radio announcements; stocking of key foods; shelf labels, cooking demonstrations; printed materials^c^; 2.5 months
Design	Prospective measurement with comparison group
Main outcomes measured	Knowledge and beliefs; awareness and use; process measures
Effectiveness	Exposure associated with increased diabetes knowledge score; sales of select targeted foods increase; consumer exposure was moderate to high: 65% of respondents had heard half or more of the 7 radio spots, 59% had seen half or more of the newspaper ads, and 31% had seen video aired on local television; reach: 120 participants; 185 population totals
**Apache Healthy Stores (AHS) (** **46** **,** **77** **)**	
Setting, location, and year^b^	11 Intervention and 6 comparison stores, White Mountain and San Carlos Apache reservations, Arizona, 2005–2006
Activities and duration	Store managers given “all” and “minimum standard” list of food items to order and promote; shelf labels and printed materials^c^; newspaper cartoons and radio announcements; cooking demonstrations and taste tests; educational displays; 12 months
Design	Prospective measurement with comparison group
Main outcomes measured	Knowledge and beliefs; process measures; intentions
Effectiveness	No effect of intervention reported for cognitive outcomes; 1,582 contacts made with customers participating in 81 cooking demonstrations; average number of customer contacts/demonstrations was 21; reach: 5% of total population
**Zhiiwaapenewin Akino’maagewin: Teaching to Prevent Diabetes (** **26** **,** **48** **)**	
Setting, location, and year^b^	3 Small or convenience stores, 4 medium-sized stores, and 1 large supermarket in 4 communities in northwestern Ontario, Canada, 2008
Activities and duration	Stocking and labeling of more healthful foods, printed materials^c^, cooking demonstrations, taste tests; radio, cable television, newsletters, and bulletin boards for educational materials; 10 months
Design	Prospective measurement with comparison group
Main outcomes measured	Process measures; knowledge and beliefs; intentions; awareness and use
Effectiveness	Significant changes in knowledge and frequency of healthful food acquisition among intervention community respondents
**Point-of-Purchase, Pricing, Increased Availability of Healthful Foods, and Promotion and Advertising**	
**Healthy Foods Hawaii (** **45** **)**	
Setting, location, and year^b^	5 Intervention and 2 comparison stores, Oahu and Big Island, Hawaii, 2010
Activities and duration	Increased store stocking of nutritious foods, educational displays, shelf labels as educational tools, cooking demonstrations and taste tests with printed materials^c^, and involvement of local producers and distributors; 11 months
Design	Prospective measurement with comparison group
Main outcomes measured	Dietary intake; process measures; knowledge and beliefs; intentions
Effectiveness	Significant effect on caregiver knowledge and attitudes; increased Healthy Eating Index score; low to moderate exposure to intervention
**Baltimore Healthy Stores (** **17** **,** **37** **,** **78** **–** **80** **)**	
Setting, location, and year^b^	2 Intervention supermarkets and 7 Korean corner stores, East Baltimore, Maryland; 2 control supermarkets and 6 Korean corner stores, West Baltimore, Maryland, 2009–2011
Activities and duration	Designed to increase availability and sales of healthier food options in local stores; culturally relevant guidelines, nutrition education training and booklet; printed materials^c^, educational displays, and shelf labels; taste tests, incentives, and giveaways; 10 months
Design	Prospective measurement with comparison group
Main outcomes measured	Knowledge and beliefs; process measures; sales data
Effectiveness	No effect on store owner outcome expectations; weekly sales of promoted foods increased when stocking improved. Reach: 2,942 participants; 55,000 population totals
**Pricing and Promotion and Advertising**	
**The Shop Smart Game (** **43** **)**	
Setting, location, and year^b^	2 Large grocery stores, Bloomington, Minnesota, 1990
Activities and duration	Aggressively advertised Bingo-style game; flyer with coupons; winning cards entered into lottery for vouchers good at participating stores; 7 weeks
Design	Multiple measurement, no comparison group
Main outcomes measured	Awareness and use
Effectiveness	High awareness of intervention
**Supermarket Healthy Options Project (SHOP) (** **33** **,** **81** **)**	
Setting, location, and year^b^	8 Supermarkets, Lower North Island, New Zealand, 2007–2009
Activities and duration	Mailed culturally relevant nutrition education materials to participants’ homes; offered price discounts on eligible healthier food products; participants were required to use, Shop ‘N Go, the electronic handheld scanner system; 9 months
Design	Single group, same point in time
Main outcomes measured	Process measures
Effectiveness	No effect of intervention on food purchases reported; mailed recruitment efforts (73% of total recruitment efforts) were more successful than community (20%) or in-store (7%) recruitment efforts

a Strategies were categorized as point-of-purchase, pricing, promotion and advertising, increased availability of healthful foods, and combinations thereof. Any results reported on reach are included in data on effectiveness. For main outcomes measured, process measures included reach, dose, and fidelity.

b Year study was published; if several articles described the same intervention, the range of years of publication is provided.

c Printed materials included at least 2 items, such as posters, recipes, or pamphlets.

The following average scores for all 7 categories were obtained: study design suitability, 2.6 (range, 1.5–3.0), effectiveness, 1.1 (range, 0–1.8), and reach, 0.3 (range, 0–1.0) (Table 2). The overall summary score for all categories combined was 8.0 (range, 3.0–12.2).

**Table 2:** Summary of Evidence (33 interventions), by Intervention Category

Strategy/Intervention	Availability of Evidence^a^	Study Design Suitability^b^	Effectiveness^c^	Reach^d^	Summary Score^e^ (0–27)	Evidence^f^
**Point-of-purchase (** **23** **,** **32** **,** **44** **,** **52** **,** **58** **–** **61** **)**						
Points	2.0	3.0	0.5	0	7.0	Insufficient
**Point-of-purchase and pricing (** **38** **)**						
Points	1.0	3.0	0	0	3.0	Insufficient
**Point-of-purchase and promotion and advertising (** **24** **,** **25** **,** **29** **,** **34** **–** **36** **,** **39** **–** **41** **,** **49** **,** **50** **,** **53** **–** **57** **,** **62** **–** **74** **)**						
Points	3.0	2.3	1.5	0.3^g^	12.2	Sufficient
**Point of purchase, pricing, and promotion and advertising (** **27** **,** **30** **,** **31** **,** **42** **,** **75** **)**						
Points	2.0	2.3	1.8	0	8.0	Insufficient
**Point-of-purchase, increased availability of healthful foods, and promotion and advertising (** **26** **,** **28** **,** **46** **,** **48** **,** **76** **,** **77** **)**						
Points	2.0	3.0	1.3	1^h^	10.7	Sufficient
**Point-of-purchase, pricing, increased availability of healthful foods, and promotion and advertising (** **17** **,** **37** **,** **45** **,** **78** **–** **80** **)**						
Points	2.0	3.0	1.5	0.5	10.0	Sufficient
**Pricing and promotion and advertising (** **33** **,** **43** **,** **81** **)**						
Points	2.0	1.5	1.0	0	5.0	Insufficient
**All strategies combined**						
Points	2.0	2.6	1.1	0.3	8.0	Insufficient

a Scored as 1, 2, or 3 points. Intervention strategy or combination of strategies represented by 10 to 30 studies was assigned 3 points; strategy or combination represented by 2 to 9 studies was assigned 2 points; and strategy or combination represented by 1 intervention was assigned 1 point.

b Scored as 1, 2, or 3 points, according to suitability of study design to determine effectiveness (15,22). Greatest suitability (3 points) refers to studies that have concurrent comparison group and prospective measurement of outcomes. Moderate suitability (2 points) refers to all retrospective designs or studies that have multiple pre- or postmeasurements but no concurrent comparison group. Least suitability refers to before–after studies that have no comparison group or studies in which outcomes were measured in a single group at the same point in time (1 point). We did not assign zero points for study design.

c Scored as 0 to 3 points, according to effectiveness of the intervention’s main outcome measures (eg, awareness and use). For example, studies reporting a 70% to 100% increase pre- to posttest or between comparison and intervention groups in awareness or use of the intervention were assigned 3 points. Studies reporting a 26% to 69% increase received 2 points. Studies reporting a 1% to 25% received 1 point. A score of 0 was assigned if no difference in outcomes was reported between study groups (29,38,44,46,52,58,77).

d Scored as 0 to 3 points. According to the RE-AIM evaluation framework (20), reach is determined by dividing the number of intervention participants by the number of people in the targeted population. For interventions reaching 70% to 100% of the population, 3 points were assigned; for 26% to 69%, 2 points; for 1% to 25%, 1 point; and for 0%, 0 points.

e Summary score (range, 0–27 points) calculated by multiplying score for availability of evidence and sum of scores for suitability, effectiveness, and reach.

f Evidence classified according to summary score: insufficient (0–9 points), sufficient (10–18 points), and strong (19–27 points).

g Only 2 of 15 interventions provided this information.

h Two of 3 interventions provided this information.

Six interventions used randomization; 2 of these used POP (23,59); 1 used POP and pricing (38); 2 used POP and promotion and advertising (29,49); and 1 used pricing and promotion and advertising (33). On average, randomized interventions had fewer points for effectiveness (0.7 points [range, 0–1]) than the 27 interventions that did not use randomization (1.4 points [range 0–3]) (24–28,30–32,34–37,39–46,50,52–54,56–58).

### POP and promotion and advertising

The level of evidence for this category (24,25,29,34–36,39–41,49,50,53–57,62–74) was sufficient. Only 7 interventions reported objective store sales data (34–36,40,50,53,57). Among them, 5 (34,36,40,50,53,57) showed increased sales of featured items and 1 intervention (35) showed no change. Only 3 interventions (34,49,50) cited a theoretical model as a framework; 1 of these interventions (49,73,74) included a policy component. The intervention “1% Or Less” (57) reported that 90% of people randomly selected for a telephone survey postintervention were aware of the campaign. The 5 A Day program’s use of audio communications was implemented as planned during the study period (49)

### POP, increased availability of healthful foods, and promotion and advertising

The level of evidence found for this category (26,28,46,48,76,77) was sufficient. In these interventions, staff worked closely with community members to determine which foods contributed most to total fat and overall calories and identified culturally relevant foods to promote. Researchers also worked with food store owners and managers to stock promoted foods and then advertised these products to consumers, thus simultaneously addressing the supply and the demand sides of healthful eating. Marshall Islands Healthy Stores (28) reported high levels of consumer exposure to the mass media components. Two interventions included self-reported purchasing data for evaluation purposes and reported a positive intervention effect. Two of 3 studies reported moderate to high fidelity; Marshall Islands Healthy Stores reported on logistical difficulties with program written materials. All of these interventions drew on social cognitive theory and included assessments of knowledge, beliefs, and intentions. One intervention (46) relied also on the social marketing framework (51). All interventions targeted low-income or racial/ethnic minority populations and were tailored to the communities in which they were implemented with culturally relevant materials and messaging.

### POP, pricing, promotion and advertising, and increased availability of healthful foods

The level of evidence for this category (17,37,45,78–80) was sufficient. Baltimore Healthy Stores (79) was a feasibility trial and not intended to reach a large number of consumers; however, the intervention reached 5% of the target population. This study collected weekly data on store sales of promoted foods (37); weekly sales of promoted foods increased in intervention stores only when stocking improved. Both programs were implemented with high fidelity. Evaluation of the other intervention included self-reported food purchases of promoted foods (45). The social cognitive theory informed both interventions, and one (45) drew also on the theory of planned behavior, reporting increased caregiver food-related knowledge but not increased intentions to purchase healthful foods.

### POP, pricing, and promotion and advertising

Although this category had insufficient evidence (27,30,31,42,75), it had the highest score for effectiveness. One intervention reported on successful nationwide dissemination of supermarket tours (31,75). Two interventions referenced a theoretical model. One (30) was based on the theory of planned behavior and the other (42) on social marketing principles. Two (27,31) interventions included self-report purchasing habits.

### POP

The level of evidence for this category (23,32,44,52,58–61) was insufficient. Five (32,44,52,58,59) interventions evaluated sales of targeted items; of these, 2 (32,59) influenced some shoppers to purchase targeted foods. One intervention (59) was based on social cognitive theory, and another (58) relied on the knowledge-attitude-performance model of behavioral change.

### Pricing and promotion and advertising

The level of evidence for this category (33,43,81) was insufficient. One intervention’s (43) evaluation relied on customer self-report food purchasing data, and the other (33) reported on participant recruitment.

### POP and pricing

The level of evidence for this category (38) was insufficient. Self-reported shopping and dietary habits (fruit and vegetable intake) were the primary outcomes, but the authors were unable to systematically compare these data between intervention and comparison stores because of inconsistent monitoring. The intervention was based on the consumer information processing model.

## Discussion

The average level of evidence for the interventions summarized in this review nears a sufficient designation but was insufficient overall. Our review generated 4 main findings. First, demand-side interventions (ie, those using POP or promotion and advertising strategies) represented most of the evidence for the study period. Second, evaluation of food store interventions in the early literature emphasized awareness of the interventions, use of the interventions, or both among target populations while excluding other important measures. Store-based strategies evolved to address the supply side, using such strategies as pricing (27,43) and increased availability of healthful foods (28,46,76,37), while continuing to increase demand by using the 2 previous approaches (POP and promotion and advertising). Innovative supply-side interventions were mostly implemented in smaller stores and could be part of a strategy for working in multiple aspects of the food environment. Reporting on process measures such as dose and fidelity is an additional strength of these interventions, allowing for elucidation of the most active components of an intervention. The transition from demand- to supply-side strategies suggests maturation in public health planning and evaluation increasingly based on social and behavioral theoretical models and addresses barriers some communities face when trying to access healthful foods. In communities with limited access to healthful foods, combining culturally sensitive demand- and supply-side strategies is effective in promoting positive food-related behaviors. Third, our review suggests that mass media campaigns accompanying POP interventions (57,70–72) have been effective population-level approaches to influence consumers’ decisions on purchasing lower-fat beverages. Finally, the limited use of randomization in food store intervention design reflects the difficulties inherent in applying this design to community-based health promotion interventions and the greater suitability of quasi-experimental designs.

We found limited evidence on the effect of the interventions on customer purchasing behavior. Eight (34,36,37,40,50,53,57,59) of 13 interventions collecting store sales data demonstrated an increase in targeted product purchases, and 13 interventions presented self-reported data on purchasing behaviors. One of these, a 12-week child-focused intervention (50) yielded a significant increase in the proportion of sales of featured items to total store sales. The intervention displayed fruit, vegetable, and healthful snack samples in a low-to-the-ground kiosk. Similarly successful interventions targeting low-income populations and drawing on social cognitive theory (46) and social marketing (79) were more likely to include components such as taste tests and focus on purchasing of more healthful items, fruit and vegetable acquisition, and food preparation.

Strengths of our study include the up-to-date systematic analysis of 58 articles identified through a comprehensive database and consideration of previous reviews (4,9–11,82). In a departure from an earlier review (4), we assessed each intervention by strategy or combination of strategies used, and we developed new categories to describe the simultaneous use of more than 1 strategy. 

Our study had several limitations. In an attempt to be comprehensive and include all evidence available to date, we included 7 studies from the late 1970s and the 1980s; data from older studies may not be relevant to today’s food environment. Findings for unsuccessful campaigns are less likely to be published in publications searchable in PubMed. We searched for published studies rather than nonpublished reports or gray literature because published articles tend to have more standardized information on setting, study design, evaluation methods, and results. We searched PubMed only. Highly controlled marketing experiments (83–87) may offer additional insights on effective strategies, but we did not include them because of their less direct translation into community-based interventions. Systematically assessing effectiveness was challenging because of the diversity in community-based interventions; many of the studies were conducted in other settings, such as small stores (37) and schools (48). Only 1 reviewer classified and scored the intervention; the classification and scoring were not verified by a second reviewer. In assessing study design, we did not differentiate between studies that used randomization and studies that did not use a control group. The differences in effectiveness suggest our estimates on levels of evidence may overestimate the actual effectiveness of food store interventions, because some of the results we assessed may have reflected baseline differences between treatment and control groups beyond the interventions implemented. Finally, because the availability of evidence was calculated as 1 of 2 subscores, newer intervention strategies will, by default, given our methods, have less research data available. Yet, some of these may offer promise given the quality of the evidence and their significant results (42,46,79).

This review focused on supermarket and grocery store interventions. Food store interventions represent only 1 level of approach among many levels — from the individual to policy (6). Increasingly, public health agencies such as the Centers for Disease Control and Prevention (88) are encouraging local communities to incorporate policy-level approaches to improve access to healthful foods (89). Task forces (90) and state and local food policy councils (91,92) have been proposed as critical elements of such efforts. These organizations leverage public incentives to help obtain financing through such mechanisms as tax exemptions, Community Development Block Grants, state grants such as the Pennsylvania Fresh Food Financing Initiative (93) (upon which Healthy Food Financing Initiative federal efforts have been based), and loans to supermarkets in underserved communities. They also ensure that funded stores participate in state food assistance programs (89,92). State and local government can, among other activities, expedite approval processes to stimulate supermarket development or encourage pedestrian-friendly development to help patrons avoid transportation barriers (89,92). Neighborhood retail analysis, incentives for energy-efficient equipment and systems, and incentives for locally grown products are other policy approaches.

Practitioners need access to up-to-date evidence when approaching grocery and supermarket owners or managers to implement interventions. Food stores want practical strategies that will change consumer behavior (94); they also need a return on investment for increasing access to more healthful foods. Some in-store efforts such as 500 Club and Footsteps to Health (www.getactivelacrosse.org/lacrosse/) complement larger environmental change, and policy initiatives (www.healthinpractice.org/ and SOS Shopping Matters), such as nutrition benefit interventions (eg, Supplemental Nutrition Assistance Program Education enacted by Healthy, Hunger-Free Kids Act of 2010) to emphasize obesity prevention as well as nutrition education and are consistent with the socioecological model (6,7), which posits that multilevel interventions addressing the connections between people and their environments maximize the effect of interventions at each level.

Our systematic review of supermarket and grocery store interventions to promote healthful eating suggests that interventions combining demand- as well as supply-side strategies have sufficient evidence to influence customers and management toward more healthful food purchases. The most effective strategies should be combined, and more rigorous evaluation designs should be used. Recent reports of the relationship between the food environment and health outcomes provide impetus for interventions to target food deserts (95–99) and represent an opportunity to add to evidence (12). Consistent with the socioecological model, public health practitioners are encouraged to use multilevel interventions, including policy and environmental change strategies, and to examine health outcomes during evaluation of these interventions.
