# Erratum, Vol. 10, April 11, 2013 Release

**DOI:** 10.5888/pcd11.120156e

**Published:** 2014-05-15

A correction was made to a sentence in the Methods section of the article “Supermarket and Grocery Store–Based Interventions to Promote Healthful Food Choices and Eating Practices: A Systematic Review.” The sentence describes the calculation of a subscore: “We used this average as 1 of 2 subscores” was changed to “We used the sum of these averages as 1 of 2 subscores.” The correction was made to our website on April 30, 2014, and appears online at http://www.cdc.gov/pcd/issues/2013/12_0156.htm. We regret any confusion or inconvenience this error may have caused.

